# Dissecting morphological changes during floral abscission in *Arabidopsis thaliana*

**DOI:** 10.1093/aobpla/plag023

**Published:** 2026-05-25

**Authors:** Risham Osahan, Natalie Hoffmann, Shelley R Hepworth

**Affiliations:** Department of Biology, Carleton University, 1125 Colonel By Dr, Ottawa, ON K1S 5B6, Canada; Department of Biology, Carleton University, 1125 Colonel By Dr, Ottawa, ON K1S 5B6, Canada; Boyce Thompson Institute, 533 Tower Rd, Ithaca, NY 14853, United States; Department of Biology, Carleton University, 1125 Colonel By Dr, Ottawa, ON K1S 5B6, Canada

**Keywords:** lignin, cellulose, morphology, floral development, abscission

## Abstract

Plant organs are shed through a tightly regulated process called abscission, which involves coordinated changes that enable cell separation and formation of a new protective barrier. However, the cellular processes within the abscission zone, where plant organs detach, are largely understudied. This gap is mainly due to difficulties in visualizing this region, due to its small size and location often being obscured by other plant tissues. Here, we present a simple and accessible method to visualize changes in cell shape and lignification in the abscission zone of *Arabidopsis thaliana* floral organs. Cell walls are stained with calcofluor white and lignin is simultaneously detected using its intrinsic autofluorescence. This inexpensive approach allows clear imaging of cellular changes during abscission zone development and can be applied to mutants with abscission-related defects.

## Teaser

We developed a rapid microscopy protocol to quantify cell shape and lignin deposition in separating sepals of Arabidopsis thaliana. This procedure will facilitate studying cellular changes occurring during organ abscission.

## Introduction

Abscission is a process by which plant organs detach in response to environmental or developmental stimuli (Roberts *et al.* 2002). Organ separation is preceded by morphological and compositional changes at the sites of plant organ boundaries, called abscission zones (AZs) (Pautot *et al.* 2025). Detachment is achieved by secretion of hydrolytic enzymes that modify the cell wall to enable cell–cell separation. During organ loss, the cells of the AZs are split into residuum cell layers, which remain on the plant body, and secession cell layers, which are attached to the abscised tissue (Lee *et al.* 2018, Pautot *et al.* 2025). In *Arabidopsis thaliana* (Arabidopsis), fertilization triggers the senescence and abscission of the floral organs, including sepals, petals, and stamens (Roberts *et al.* 2002). Despite its ubiquitous occurrence, the cellular changes that occur in floral AZs remain poorly understood. This is largely because AZs are obscured by surrounding organs prior to separation, making them difficult to visualize. Consequently, imaging floral AZs requires specialized methods distinct from other tissues.

Extensive studies have shown that development of AZs and floral abscission in Arabidopsis follows a highly regulated pattern (Bleecker and Patterson 1997, McKim *et al.* 2008, Pautot *et al.* 2025). Flowers can be numbered by developmental stage or position, with the youngest open flower designated as position 1 (P1) and progressively older flowers numbered sequentially (Bleecker and Patterson 1997). Scanning electron microscopy has been used to characterize AZ morphology at different positions (Bleecker and Patterson 1997, Shi and Butenko 2018) focusing primarily on residuum cells before, during, and after organ detachment. Morphological changes in secession cells can also inform on the progression of abscission but have not been thoroughly described in a developmental context.

A phenolic polymer called lignin accumulates in the cell walls of secession cells at the base of separating organs (Lee *et al.* 2018, Crick *et al.* 2022). Within these cell walls, lignin acts to reinforce the tissue during mechanical detachment and to confine the hydrolytic enzymes secreted by the adjacent residuum layer (Lee *et al.* 2018). Lignification begins at the corners of the cell wall before extending to surrounding cell wall regions, forming a honeycomb-like lignin brace structure. This patterned deposition of lignin may result from the specific cell corner localization of laccase and peroxidase enzymes involved in its polymerization (Lee *et al.* 2018, Hoffmann *et al.* 2020). Two NADPH oxidases, RBOHD and RBOHF, provide the H_2_O_2_ required for these peroxidases and are essential for AZ lignification (Lee *et al.* 2018).

The amount, timing, and organization of lignin deposition are involved in abscission in many plant species, including pepper (*Capsicum annuum*; Hill *et al.* 2023), *Brachypodium distachyon* (Yu *et al.* 2020a), and rice (*Oryza sativa*; Wu *et al.* 2023). In Arabidopsis, the lignin brace is not necessary for floral abscission. However, mutations in organ boundary genes such as *BLADE-ON-PETIOLE* (*BOP*) or the three-amino-acid-loop-extension (TALE) homeodomain transcription factor *ARABIDOPSIS THALIANA HOMEOBOX GENE1* (*ATH1*) impair lignin deposition and exhibit absent or delayed abscission (Hepworth *et al.* 2005, Norberg *et al.* 2005, Gómez-Mena and Sablowski 2008, Crick *et al.* 2022). Therefore, the specific role of the lignin brace in Arabidopsis is still unclear and requires further study.

Common methods for visualizing lignin include phloroglucinol-HCl (Weisner) and Mäule staining. Phloroglucinol-HCl staining has been widely used to detect lignin in Arabidopsis tissues including AZs (Mitra and Loqué 2014, Shafi *et al.* 2015, Herrera-Ubaldo and de Folter 2018, Lee *et al.* 2018). However, its pink colouration fades quickly, making it unsuitable for long-term sample preservation. It also requires the use of concentrated HCl, which is corrosive and requires safe handling (Mitra and Loqué 2014). Mäule staining is another widely used method for lignin detection (Mitra and Loqué 2014, Shafi *et al.* 2015), but this too involves corrosive ammonium hydroxide and samples require immediate imaging.

An alternative approach avoids staining altogether by exploiting the intrinsic autofluorescence of lignin under ultra-violet (UV) fluorescent light. UV illumination excites aromatic components of the lignin polymer, providing bright autofluorescence (Donaldson 2013). This intrinsic signal can be used to assess the spatial distribution of lignin and to quantify its relative abundance across developmental stages or different tissues (Mitra and Loqué 2014, Lee *et al.* 2018, Hoffmann *et al.* 2020).

In addition to lignification, AZ cells undergo morphological changes in size and shape (Crick *et al.* 2022). Methods for mapping these changes mostly focus on imaging polysaccharide components of the plant cell wall, such as cellulose, hemicellulose, and pectin. Congo red and calcofluor white are two commonly used dyes that bind to cellulose (Herrera-Ubaldo and de Folter 2018). Congo red is used less frequently due to its carcinogenic and mutagenic properties (Kitin *et al.* 2020). In contrast, calcofluor white is a safe, inexpensive, and efficient alternative that enables the rapid visualization of cell shape in a wide range of plant tissues, including AZs (Mitra and Loqué 2014, Crick *et al.* 2022).

Here, we present a simple method for the combined visualization of lignin and cellulose in cleared Arabidopsis sepal AZs using epifluorescence microscopy of intrinsic lignin autofluorescence with calcofluor white staining of cell walls. We show how this approach can be used qualitatively and quantitatively to study cell shape and lignin deposition during developmental floral abscission and how these processes are altered in mutant backgrounds with abscission-related defects.

## Materials and methods

### Plant materials and growth conditions

*Arabidopsis thaliana* plants were grown on soil in ATC60 growth chambers (Conviron, Winnipeg, Canada) at 21°C under long-day conditions (16 h light/8 h dark) with a light intensity of ∼115 μmol m^−2^ s^−1^. Seeds were surface sterilized in 3% sodium hypochlorite (bleach) and 0.5% SDS (ThermoFisher Scientific, Ottawa, Canada) and sown directly on autoclaved soil (Promix BX, Premier Horticulture, Rivière-du-Loup, Canada) supplemented with 20-20-20 fertilizer (1 g L^−1^; Plant-Prod Inc, Brampton, Canada) and Plant Prod Chelates Micronutrient fertilizer (0.063 g L^−1^; Plant-Prod Inc., Brampton, Canada). Seedlings were transplanted into 72-cell propagation trays and grown to maturity. All lines were in the Columbia-0 (Col-0) background, which served as the wild-type control. Mutants used were: *bop1-6D*, an activation-tagged line that overexpresses *BOP1* (Norberg *et al.* 2005), T-DNA insertion mutant *ath1-3* (SALK_113353; Gómez-Mena and Sablowski 2008), and transposon mutant *rbohD rbohF* (Torres *et al.* 2002). These mutants exhibit distinct AZ-related phenotypes: *bop1-6D* shows growth defects and early abscission (Norberg *et al.* 2005, Crick *et al.* 2022); *ath1-3* shows delayed and incomplete abscission (Gómez-Mena and Sablowski 2008, Crick *et al.* 2022); and *rbohD rbohF* lacks the lignin brace in secession layers (Lee *et al.* 2018).

### Abscission scoring

Abscission was scored along the primary stem using at least 24 plants per genotype. Position 1 (P1) designates the first open bud, with each subsequent flower named sequentially (Bleecker and Patterson 1997). Initiation of abscission was defined as the position at which organs start to detach with gentle mechanical touch (Crick *et al.* 2022).

### Sample preparation

Sepals were collected from mature abscising plants, approximately 5–6 weeks old. Whole flowers were cut from the stem using sharp forceps, beginning at P1. Under a dissecting microscope, the floral tip near the stigma was gently held in place with the index finger of the non-dominant hand. Using forceps with the dominant hand, the sepal was slowly pulled downward towards the receptacle until it detached. Care was taken not to damage the flower. Detached sepals from a given developmental stage were placed in a microcentrifuge tube containing 70% ethanol and were stored at room temperature overnight or until the tissue was cleared. This procedure was repeated for flowers at all pre-abscission positions (typically P0–P6). Due to the slightly earlier abscission of *rbohD rbohF*, late P5 sepals were classified as P6 stage for comparison purposes, as the last stage prior to organ loss.

Abscised cauline leaves from 8-week-old *Camelina sativa* plants were processed as described above. Siliques from 8-week-old Arabidopsis Col-0 plants were fixed and embedded in Paraplast Plus (Sigma Aldrich, St. Louis, Missouri, USA) from which 10 µm sections were cut using a microtome, affixed to glass slides (Superfrost Plus, Fisher Scientific, Toronto, Canada) and dewaxed with xylene prior to staining.

### Lignin autofluorescence and calcofluor staining

Sepal samples were further cleared using a protocol modified from DiLaurenzio *et al.* (1996) and Malamy and Benfey (1997). 70% ethanol was replaced with 0.24 N HCl in 20% methanol, and samples were incubated in a 57°C water bath for 90 min. Cleared sepals were then treated with 7% NaOH in 60% ethanol for 15 min at room temperature, followed by a graded ethanol rehydration series (40%, 20%, and 10%) for 5 min each.

A 10% w/v stock solution of calcofluor white (PhytoTech; catalog #C1933) was prepared in distilled water, protected from light, and stored at 4°C. A 0.1% w/v working solution was freshly prepared on the day of use. Sepals were incubated in 0.5 ml of staining solution for 5 min in the dark, then rinsed five times with distilled water to remove excess dye.

Stained sepals were equilibrated in 25% glycerol in 5% ethanol and mounted in 50% glycerol on microscope slides. A coverslip was placed over the samples and the edges were sealed with clear nail polish to prevent drying. Slides were stored in the dark until imaging. Experiments were conducted between January 2023 and August 2024, and slides were re-imaged in September 2025. Leaf and silique tissue was stained and imaged as described above in April 2026.

### Fluorescence imaging

Fluorescence imaging was performed using a Zeiss AxioImager M2 microscope (Carl Zeiss Canada Ltd., Toronto, Canada) equipped with a Zeiss Colibri LED light source and a Zeiss AxioCam digital camera. Samples were imaged using a 20× Plan-APOCHROMAT objective (0.8 N.A., 0.55 mm W.D.). Image acquisition and initial processing were performed using ZEN microscopy software (Carl Zeiss Canada Ltd., Toronto, Canada). For lignin autofluorescence, samples were excited at 470 nm and observed using the 62 HE fluorescence filter set (BP 370/40 nm, 474/28 nm, and 585/35 nm). For calcofluor white fluorescence, samples were excited at 365 nm and observed with the same filter set. Imaging was conducted on 16–30 sepals from 4 to 8 biological replicates in three independent experiments, all yielding consistent results. Each biological replicate represents a flower from a different plant.

### Image processing and visualization

Images were aligned and slightly sharpened around the edges using Adobe Photoshop 2023 (version 25.2; Adobe Inc., San Jose, USA). Calcofluor white images are displayed in greyscale, with black and grey pixels denoting stained cell walls. Lignin autofluorescence is shown in cyan. Heat maps of lignin intensity were generated in FIJI (Schindelin *et al.* 2012) using the *Fire* Look Up Table.

### Quantification of lignin autofluorescence

Lignin autofluorescence was quantified following Hoffmann *et al.* (2020) in FIJI (Schindelin *et al.* 2012). Identical microscope settings were used for all samples. For each sepal, lignin autofluorescence within the AZ was measured from three 100 × 100-pixel regions of interest. Within the same image, three 100 × 100-pixel regions were selected in adjacent non-AZ tissues to calculate the mean background fluorescence intensity. AZ fluorescence values were corrected by subtracting and dividing by the mean background value. Corrected AZ fluorescence values were then averaged to yield a single fluorescence value per sepal (*n* = 5–8 sepals per genotype). Averages were compared using a one-way ANOVA with a non-parametric Dunn’s *post hoc* test with Bonferroni correction.

## Results and discussion

**Figure 1:**
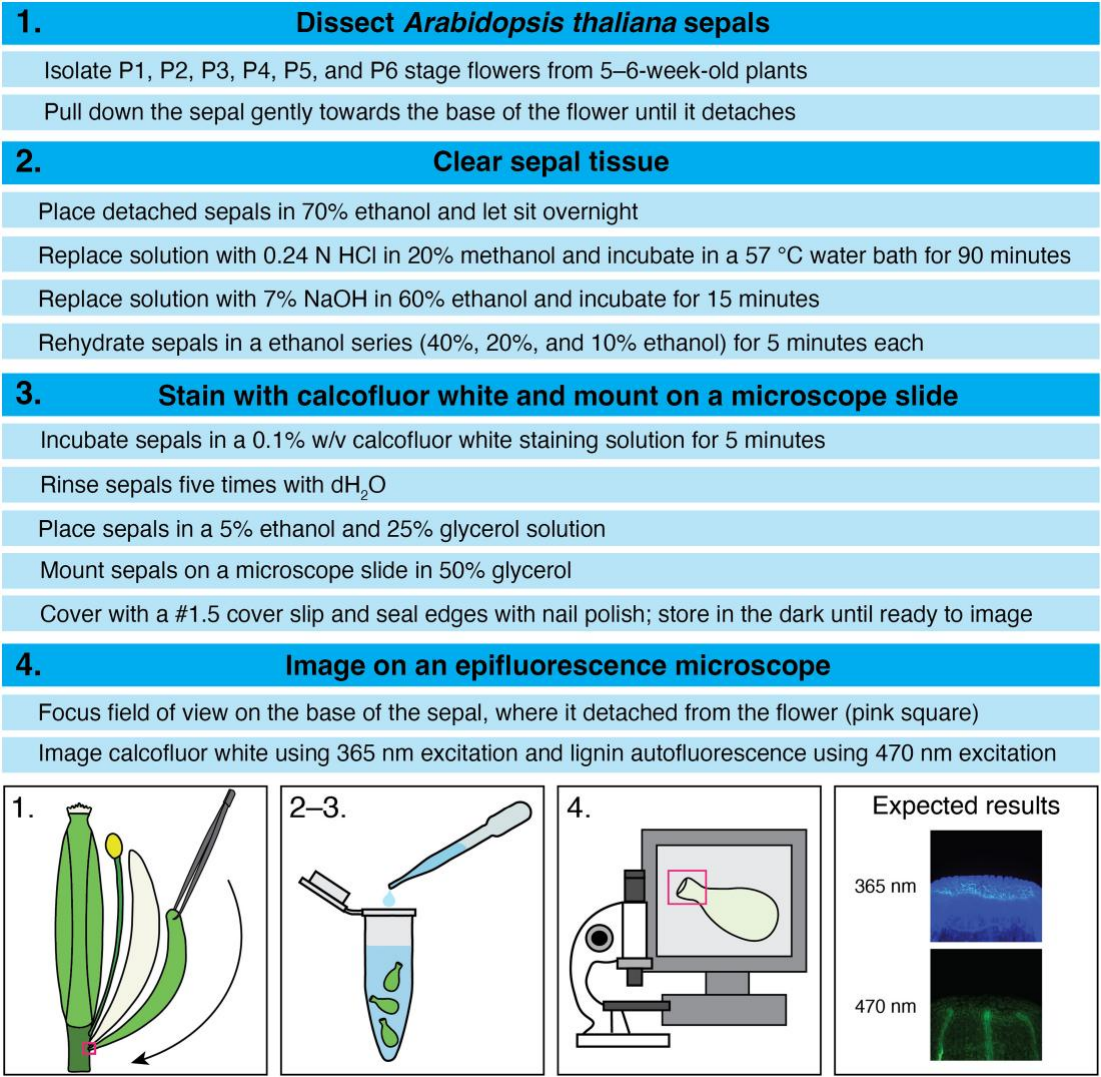
Dual imaging workflow for sepal AZ analysis. Steps include: (1) isolation of Arabidopsis sepals, (2) tissue clearing to remove chlorophyll and other autofluorescent compounds, (3) calcofluor white staining, and (4) epifluorescence imaging. The basal region of the sepal, corresponding to the secession cells of the AZ, was imaged to visualize cell wall architecture (calcofluor white) and autofluorescence (lignin).

We developed a simple imaging method to simultaneously visualize cell morphological changes and lignin deposition in detaching organs during floral abscission. A schematic overview of the sample collection, staining, and imaging workflow is shown in Fig. 1. The protocol combines stain-free detection of lignin, through its broad intrinsic autofluorescence, with calcofluor white staining to label plant cell walls. Calcofluor white has an excitation maximum at 340–380 nm and emission between 430 and 475 nm, whereas lignin autofluorescence can be detected using 470 nm excitation and 500–575 nm emission (Donaldson 2013), allowing clear separation of the two signals. To evaluate the method, we studied wild-type (Col-0) flowers and mutants with known abscission-related defects, including *bop1-6D* (Norberg *et al.* 2005), *ath1-3* (Gómez-Mena and Sablowski 2008), and *rbohD rbohF* (Lee *et al.* 2018).

**Figure 2:**
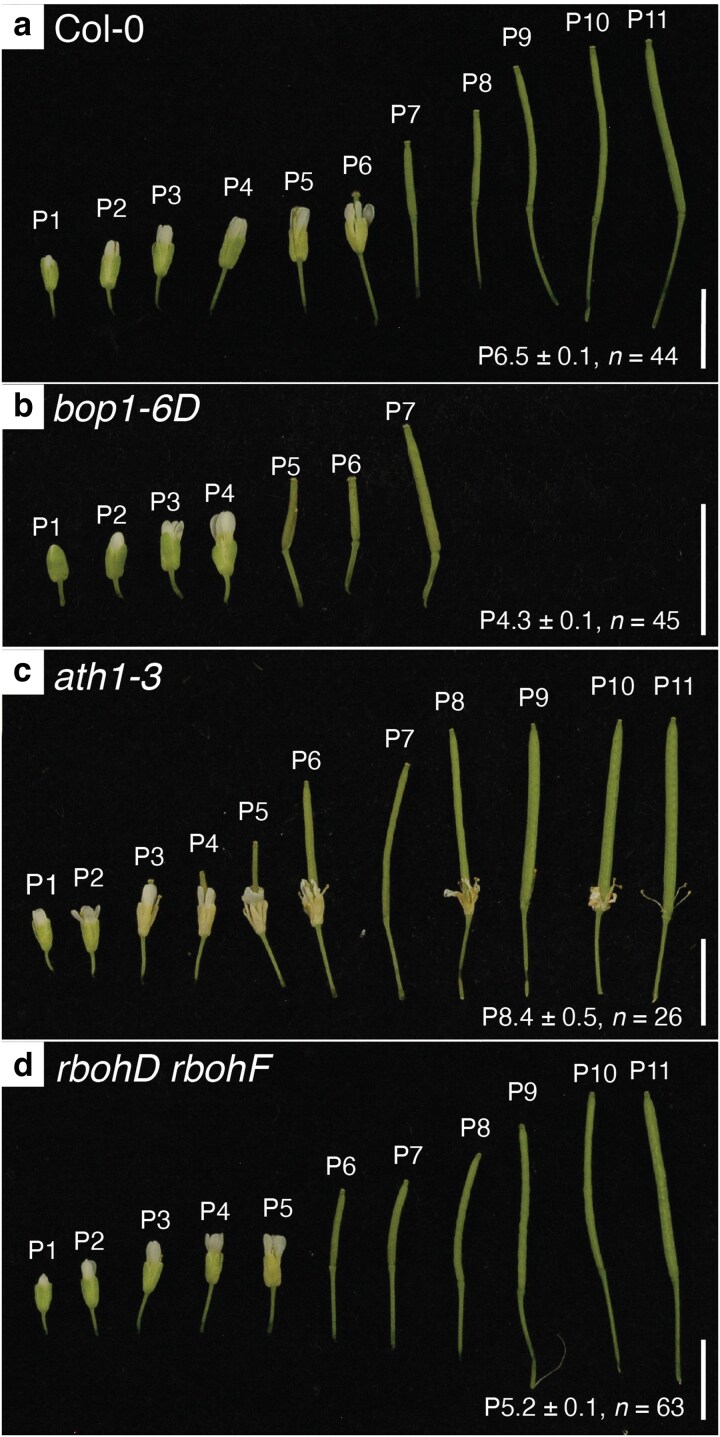
Developmental series of floral organ abscission in wild-type and mutant Arabidopsis. The first open bud was defined as position 1 (P1) (Bleecker and Patterson 1997). Abscission position (mean ± SEM) is indicated in the lower right of each panel. Representative phenotypes are shown. (a) Wild-type Col-0 flowers abscise between P6 and P7. (b) *bop1-6D* exhibits early abscission. (c) *ath1-3* shows delayed abscission and retention of floral organs. (d) *rbohD rbohF* shows slightly accelerated abscission. Scale bars = 5 mm.

We first examined AZ development and floral organ shedding in Col-0 (Fig. 2a). Consistent with previous studies (Bleecker and Patterson 1997, McKim *et al.* 2008), floral organ abscission occurred at P6.5 ± 0.1 (mean ± SEM; *n* = 44 flowers). Overexpression of *BOP1*, a key regulator of AZ initiation, led to shorter stature and precocious abscission by P4.3 ± 0.1 (mean ± SEM; *n* = 45 flowers) (Norberg *et al.* 2005, Crick *et al.* 2022, Fig. 2b). In contrast, mutation of *ATH1*, which contributes to AZ boundary formation, showed irregular abscission at P8.4 ± 0.5 (mean ± SEM; *n* = 26 flowers), with stamens remaining attached through P11 (Gómez-Mena and Sablowski 2008, Fig. 2c). The *rbohD rbohF* double mutant, which lacks NADPH oxidases required for lignin polymerization, showed slightly earlier abscission relative to Col-0 at P5.2 ± 0.1 (mean ± SEM; *n* = 63 flowers) (Fig. 2d), consistent with previous work (Crick *et al.* 2022, Lalun *et al.* 2024).

**Figure 3:**
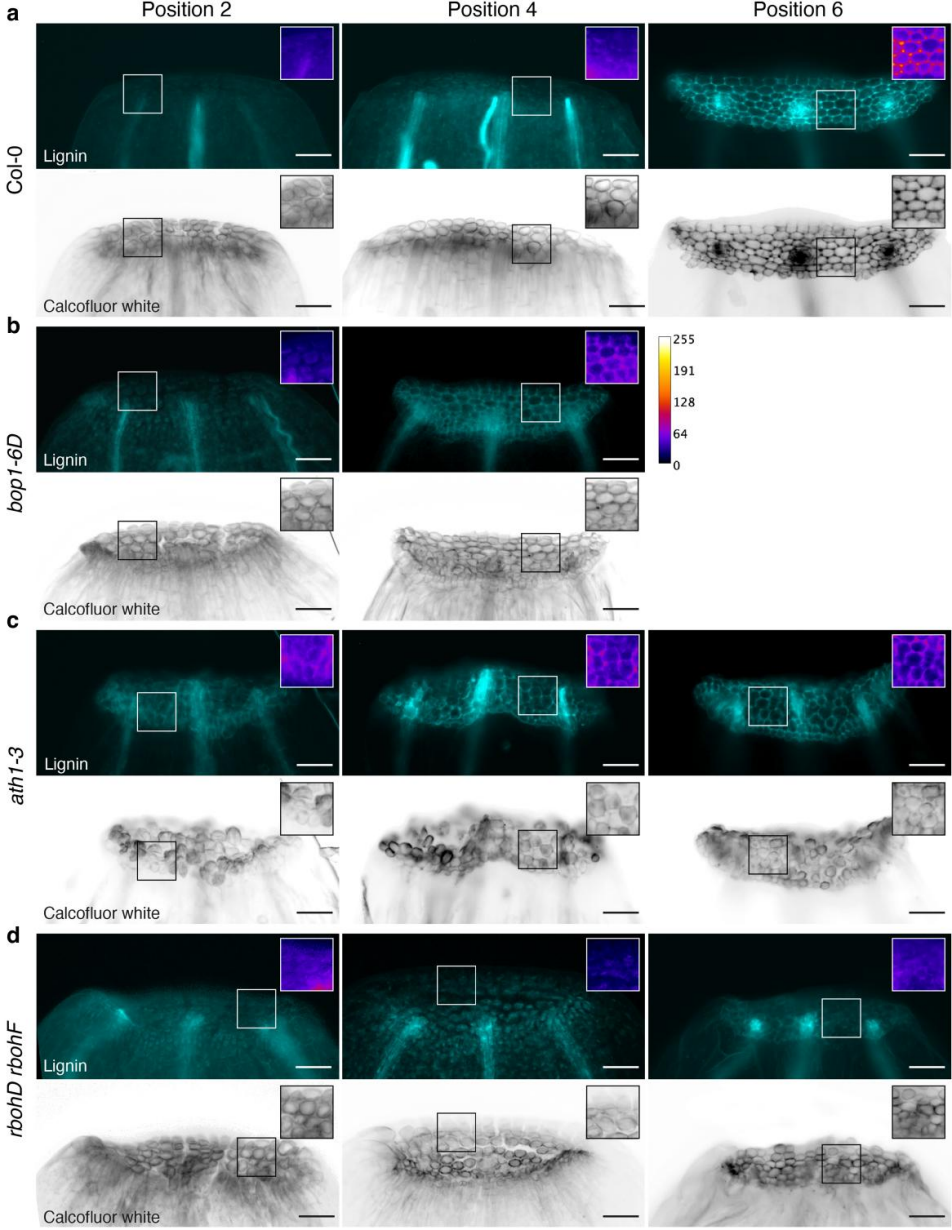
Dual imaging of lignin autofluorescence and cell wall architecture in sepal AZs. Sepal AZs at P2, P4, and P6 were imaged for lignin autofluorescence (cyan; upper panels) and calcofluor white staining (grey scale; lower panels). Boxed regions are magnified in the upper right (white boxes, lignin; black boxes, calcofluor white). Insets of lignin autofluorescence are displayed as heatmaps to show relative fluorescence intensity. The intensity scale is consistent across all panels (shown next to b). (a) Wild-type Col-0 shows a clearly defined brace at P6. (b) *bop1-6D* shows early lignification and associated cell shape changes at P4. (c) *ath1-3* shows disordered early lignification and abnormal cellular organization at all stages. (d) *rbohD rbohF* lacks a lignin brace and associated cell shape changes. Scale bars = 25 μm.

We focused our imaging on sepal AZs because they are larger and more easily visualized compared to petal or stamen AZs. Sepals from P2, P4, and P6, as representative stages leading up to Col-0 abscission, were dissected, cleared, and imaged for lignin autofluorescence and calcofluor white staining (Fig. 3). In Col-0, lignin autofluorescence was absent at P2 or P4, but clearly visible at P6 (Fig. 3a), first appearing at P5 for most samples. This pattern indicates that lignin deposition precedes organ separation, consistent with transcriptomic evidence showing upregulation of lignin biosynthesis genes in secession cells prior to abscission (Taylor *et al.* 2024). Fluorescence intensity heatmaps confirmed the absence of lignin signal in cell walls in early stages (P2 and P4) but strong accumulation by P6, particularly in cell corners (Fig. 3a, insets). Calcofluor white staining revealed corresponding changes in cell shape during AZ formation. At P2 and P4, Col-0 AZs cells were rounded, but by P6, lignin brace formation was accompanied by the development of rigid hexagonal cells, with thicker cell walls relative to earlier positions (Fig. 3a). In the *bop1-6D* overexpression line, which exhibits early abscission (Fig. 2b) imaging was limited to P2 and P4. The lignin brace was absent at P2 but appeared earlier than in Col-0, and was fully formed by P4 (Fig. 3b). Calcofluor white imaging showed that cell morphology transitioned from round at P2 to rigid and angular by P4 (Fig. 3b). These results indicate that *BOP1* overexpression accelerates the normal sequence of cellular events during abscission without altering their overall pattern. The *ath1-3* mutant exhibits disordered AZ formation characterized by abnormal lignification (Crick *et al.* 2022). Using the dual imaging method, spotty lignin autofluorescence was visible as early as P2 and P4 (Fig. 3c). Lignin is deposited unevenly around cells of varying sizes. Calcofluor white staining further revealed that AZ cells were abnormally arranged at all developmental stages, drawing attention to disrupted cellular organization within the sepal AZ (Fig. 3c). The observations support an essential role for ATH1 in patterning the AZ cell layers (Crick *et al.* 2022), which is necessary for the different cellular changes that occur between abscission layers. In contrast, the *rbohD rbohF* double mutant, deficient in NADPH oxidases required for H_2_O_2_-dependent peroxidase activity (Lee *et al.* 2018), showed no lignin deposition at any stage (Fig. 3d). The overall AZ organization appeared similar to Col-0, but lacked the thickened walls and hexagonal cell shapes typical of lignified tissues, indicating that these structural features depend on proper lignin deposition but are not necessary for abscission.

**Figure 4:**
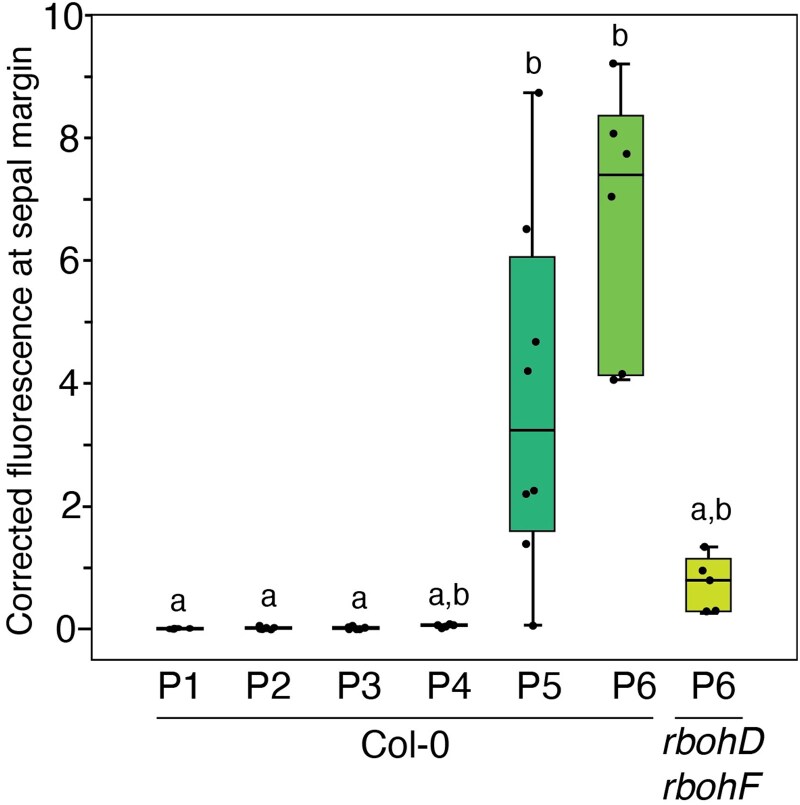
Graph showing relative amount of lignin autofluorescence in sepal AZs. Fluorescence intensity was measured in Col-0 sepals from P1–P6 and *rbohD rbohF* sepals at P6. Each data point represents one sepal (*n* = 5–8 sepals per developmental stage or background). Differences among groups were assessed using a one-way ANOVA followed by Dunn’s *post hoc* test with Bonferroni correction. Different letters indicate statistically different averages, *P* < 0.02.

To support the qualitative observations of lignin deposition during AZ development, we quantified lignin autofluorescence using a protocol previously applied to Arabidopsis stem development (Hoffmann *et al.* 2020). All images were captured using identical microscope settings and fluorescence intensity was calculated for each developmental stage after background correction. In Col-0 AZs, lignin signal first appeared at P5 and increased markedly by P6, whereas lignin-deficient *rbohD rbohF* AZs showed minimal fluorescence (Fig. 4). Interestingly, *rbohD rbohF* exhibited a background of weak, diffuse fluorescence relative to earlier Col-0 stages. Although the source of this signal is unclear, it may reflect the accumulation of unpolymerized lignin monomers or other phenolics (Lee *et al.* 2018, Crick *et al.* 2022). Together, these approaches provide both qualitative and quantitative insight into AZ structure and composition, offering a valuable tool for investigating the cellular mechanisms of abscission. In particular, it highlights the sensitivity of the autofluorescence quantification for identifying subtle variations in lignin phenotypes.

This protocol uses epifluorescence microscopy, which offers a rapid and accessible approach for lignin imaging in most laboratory settings and provides sufficient cellular resolution for quantitative analysis (Mitra and Loqué 2014, Kitin *et al.* 2020). However, it lacks the optical sectioning and depth resolution available with confocal microscopy, which can also be used for this protocol but is more time-consuming and costly to operate. Epifluorescence microscopy remains well-suited for thick, three-dimensional plant tissues like the sepal, compared to confocal microscopy techniques that view samples at a single focal plane and require extended acquisition times to image different focal planes required for three-dimensional datasets.

While this protocol allows rapid screening of changes in AZ morphology between different mutant backgrounds, it does not provide mechanistic information about how these observed changes impact abscission. Rather, this methodology should be used as a first step to identify developmental stages or mutant lines that can be studied with further experiments. In the lignin autofluorescence images, some background signal is detected outside the AZ, primarily from lignin-rich xylem vessels (visible as vertical lines) and other autofluorescent cellular components (Fig. 3). Nevertheless, the lignin brace is clearly distinguishable by its characteristic hexagonal structure, which appeared at the developmental stage just prior to organ abscission (Fig. 3a, inset). The highly reduced autofluorescent signal in the *rbohD rbohF* mutant (Figs 3d and 4) aligns with previous reports showing the absence of a lignin brace (Lee *et al.* 2018), confirming that the signals detected using this method are specific to lignin.

The role of the lignin brace during abscission is still unclear, although it is hypothesized to be involved in mechanical support of abscising tissues and to limit the diffusion of secreted hydrolytic enzymes (Lee *et al.* 2018). Previous transcriptomic and histochemical studies have found both positive (Merelo *et al.* 2017, He *et al.* 2025) and negative (Yoon *et al.* 2014) correlations between lignin deposition and abscission in diverse plant species, and some species lack detectable lignin in their AZs (Yu *et al.* 2020b).

In this study, lignin brace formation varied across genotypes. It was accelerated in *BOP1* overexpression lines that undergo earlier abscission; had a patchy distribution in *ath1-3* mutants with abnormal, delayed abscission; and was absent in *rbohD rbohF* lines that abscised slightly earlier than Col-0 (Figs 2 and 3). Accelerated lignin deposition in *BOP1* overexpression lines is consistent with work showing that upregulation of *BOP1* promotes earlier and more extensive lignification in stems (Khan *et al.* 2012). Because lignin biosynthesis is upregulated during the AZ developmental programme (Taylor *et al.* 2024), increased *BOP1* expression, an early regulator of AZ formation (McKim *et al.* 2008), likely accelerates both lignin deposition and abscission. In contrast, the patchy lignin distribution in *ath1-3* mutants correlates with boundary defects that disrupt AZ structure and may impair responsiveness to abscission signals (Crick *et al.* 2022). The ability of *rbohD rbohF* mutants to abscise in the absence of a lignin brace indicates that lignin is not essential for floral organ abscission in Arabidopsis (Lee *et al.* 2018, Crick *et al.* 2022, Lalun *et al.* 2024). Future work is therefore needed to clarify the functional role of the lignin brace, especially in other abscising tissues or conditions where mechanical forces influence the shedding process.

In summary, currently available methods to image lignin and cell morphology often require expensive microscopes that are not readily accessible in all research environments. In addition, few imaging methodologies are tailored for imaging the unique structure of AZs. We have presented a time-friendly staining technique that can be performed using basic laboratory supplies and standard epifluorescence microscopy. This method enables the visualization of cell morphology and lignin deposition in high-resolution images in Arabidopsis sepal AZs and places these morphological changes within the broader context of developmental abscission. We showed that lignin brace formation precedes organ separation and described morphological changes in mutant lines with defects in abscission.

Furthermore, this methodology could be adapted for other lignified tissues in diverse species, such as lignin in the gynoecium (Herrera-Ubaldo and de Folter 2018), the lignified layer involved in fruit dehiscence (Ballester and Ferrándiz 2017), or anther dehiscence (Hao *et al.* 2014). As a proof of concept, we have used this imaging protocol to examine abscised cauline leaves from *C. sativa* and the dehiscence zone of siliques in Arabidopsis (see Supplementary Fig. S1). The AZ of *C. sativa* leaves has rounded cells with no obvious lignin brace. In contrast, the dehiscence zone at the valve margin and the endocarp layer *b* are well lignified, as demonstrated previously (Roeder and Yanofsky 2006). This method is therefore suitable for imaging a wide range of tissues and species.

## Supplementary Material

plag023_Supplementary_Data

## Data Availability

All relevant data and details of resources can be found within the article and Supplementary Information.
