# Supplementary material for: Dissecting morphological changes during floral abscission in Arabidopsis thaliana
Source: AoB Plants. 2026 May 25;18(3):plag023. doi: 10.1093/aobpla/plag023 (PMC13267139; doi:10.1093/aobpla/plag023)
Supplement: plag023_Supplementary_Data [file plag023_supplementary_data.pdf]

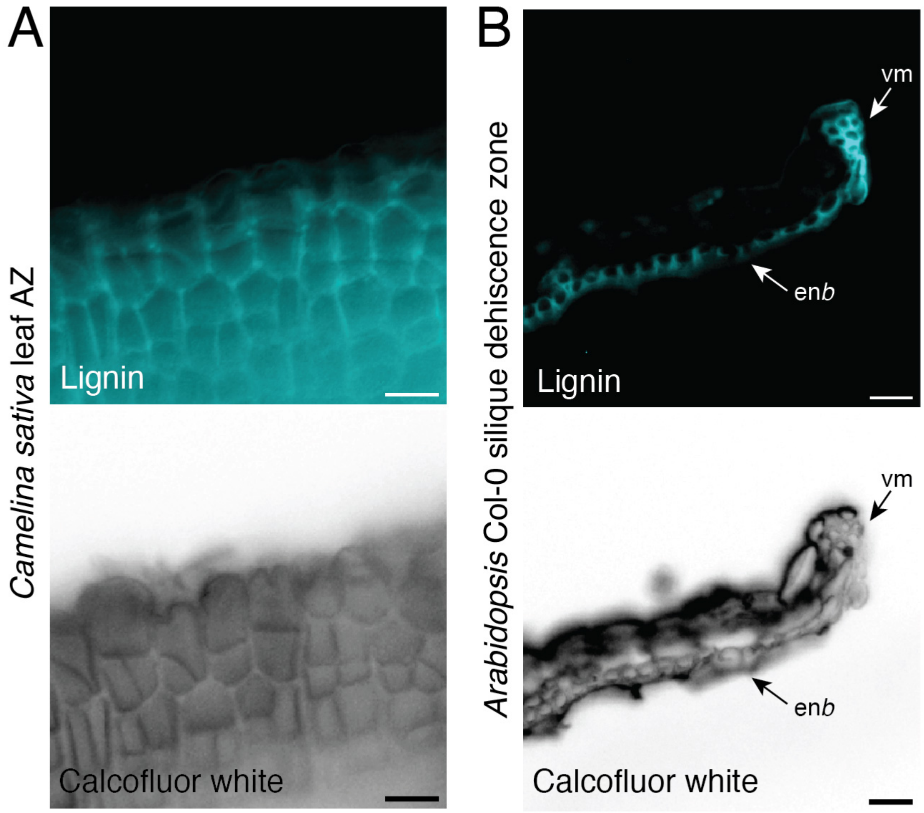

Figure S1. Dual imaging of lignin autofluorescence and cell wall architecture in other tissue types and plant species. **A)** Abscised cauline leaf tissue from *Camelina sativa*. **B)** The dehiscence zone located at the valve margin in Arabidopsis siliques. Vm indicates the lignified valve margin, en*b* indicates the lignified endocarp *b* cell layer. Images are representative of three biological replicates per tissue. Scale bars = 20 μm.

<u>Alt Text:</u> Microscope images showing lignin distribution and cell wall shape in leaf tissue of *Camelina sativa* and in the silique of Arabidopsis.
